# 2-Amino-4-methyl­pyridinium 3-chloro­benzoate

**DOI:** 10.1107/S160053681002444X

**Published:** 2010-06-26

**Authors:** Madhukar Hemamalini, Hoong-Kun Fun

**Affiliations:** a X-ray Crystallography Unit, School of Physics, Universiti Sains Malaysia, 11800 USM, Penang, Malaysia

## Abstract

In the title salt, C_6_H_9_N_2_^+^·C_7_H_4_ClO_2_^−^, the 2-amino-4-methyl­pyridinium cation is almost planar, with a maximum deviation of 0.010 (1) Å. In the crystal, the protonated N atom and the 2-amino group of the cation are hydrogen bonded to the carboxyl­ate O atoms of the anion *via* a pair of N—H⋯O hydrogen bonds, forming an *R*_2_^2^(8) ring motif. The ion pairs are further connected *via* N—H⋯O and C—H⋯O hydrogen bonds, forming a two-dimensional network parallel to the *bc* plane.

## Related literature

For details of non-covalent inter­actions, see: Remenar *et al.* (2003 ▶); Aakeroÿ *et al.* (2001 ▶); Sokolov *et al.* (2006 ▶). For related structures, see: Kvick & Noordik (1977 ▶); Shen *et al.* (2008 ▶); Hemamalini & Fun (2010**a* ▶,b* ▶). For details of hydrogen bonding, see: Jeffrey & Saenger (1991 ▶); Jeffrey (1997 ▶); Scheiner (1997 ▶). For hydrogen-bond motifs, see: Bernstein *et al.* (1995 ▶). For bond-length data, see: Allen *et al.* (1987 ▶). For the stability of the temperature controller used in the data collection, see: Cosier & Glazer (1986 ▶).
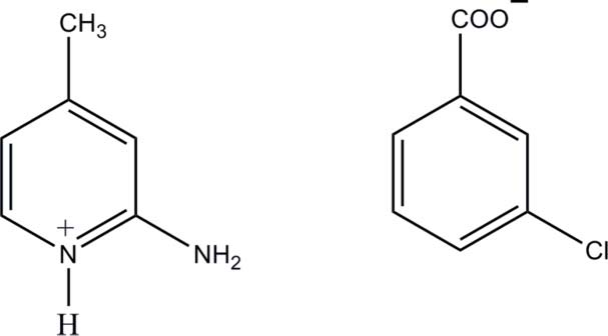


## Experimental

### 

#### Crystal data

C_6_H_9_N_2_^+^·C_7_H_4_ClO_2_^−^
*M* *_r_* = 264.70
Monoclinic, 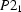

*a* = 7.9930 (6) Å
*b* = 6.8608 (5) Å
*c* = 11.2148 (9) Å
β = 93.526 (2)°
*V* = 613.84 (8) Å^3^
*Z* = 2
Mo *K*α radiation
μ = 0.31 mm^−1^
*T* = 100 K
0.28 × 0.17 × 0.10 mm

#### Data collection

Bruker APEXII DUO CCD diffractometer
Absorption correction: multi-scan (*SADABS*; Bruker, 2009 ▶) *T*_min_ = 0.919, *T*_max_ = 0.971
9325 measured reflections
4207 independent reflections
4076 reflections with *I* > 2σ(*I*)
*R*_int_ = 0.019

#### Refinement

*R*[*F* ^2^ > 2σ(*F* ^2^)] = 0.029
*wR*(*F* ^2^) = 0.117
*S* = 1.22
4207 reflections
164 parameters
1 restraint
H-atom parameters constrained
Δρ_max_ = 0.64 e Å^−3^
Δρ_min_ = −0.54 e Å^−3^
Absolute structure: Flack (1983 ▶), 1860 Friedel pairs
Flack parameter: −0.01 (4)

### 

Data collection: *APEX2* (Bruker, 2009 ▶); cell refinement: *SAINT* (Bruker, 2009 ▶); data reduction: *SAINT*; program(s) used to solve structure: *SHELXTL* (Sheldrick, 2008 ▶); program(s) used to refine structure: *SHELXTL*; molecular graphics: *SHELXTL*; software used to prepare material for publication: *SHELXTL* and *PLATON* (Spek, 2009 ▶).

## Supplementary Material

Crystal structure: contains datablocks global, I. DOI: 10.1107/S160053681002444X/hb5503sup1.cif

Structure factors: contains datablocks I. DOI: 10.1107/S160053681002444X/hb5503Isup2.hkl

Additional supplementary materials:  crystallographic information; 3D view; checkCIF report

## Figures and Tables

**Table 1:** Hydrogen-bond geometry (Å, °)

*D*—H⋯*A*	*D*—H	H⋯*A*	*D*⋯*A*	*D*—H⋯*A*
N1—H1*A*⋯O1^i^	0.86	1.83	2.6921 (16)	175
N2—H2*B*⋯O2^i^	0.86	1.93	2.786 (2)	177
N2—H2*C*⋯O2^ii^	0.86	1.96	2.8146 (14)	173
C5—H5*A*⋯O1^iii^	0.93	2.50	3.1707 (13)	129

Symmetry codes: (i) 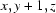; (ii) 
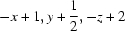
; (iii) 
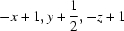
.

**Fig. 1.**
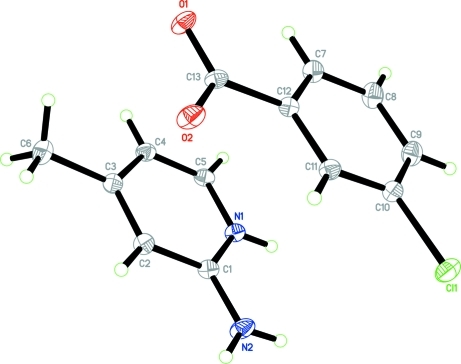
The asymmetric unit of (I). Displacement ellipsoids are drawn at the 50% probability level.

**Fig. 2.**
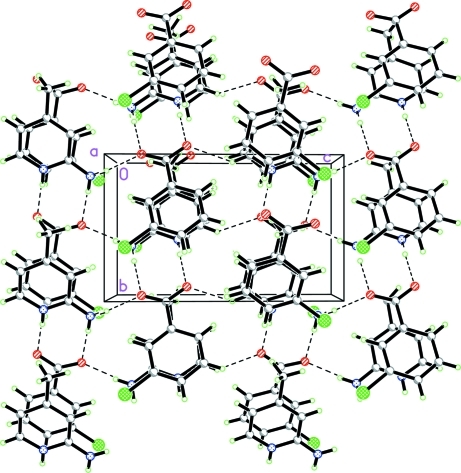
The crystal packing of (I), showing hydrogen-bonded (dashed lines) 2D networks parallel to the bc-plane.

## supplementary crystallographic information

### Comment

Recently, much attention has been devoted to the design and synthesis of supramolecular architectures assembled via various weak noncovalent interactions, such as hydrogen bonds, π···π stacking and C—H···π interactions (Remenar *et al.*, 2003; Aakeroÿ *et al.*, 2001; Sokolov *et al.*, 2006). 2-Aminopyridine and its derivatives are used in the manufacture of pharmaceuticals, hair dyes and other dyes. They are often involved in hydrogen-bond interactions (Jeffrey & Saenger, 1991; Jeffrey, 1997; Scheiner, 1997). The crystal structures of 2-amino-4-methyl pyridine (Kvick & Noordik, 1977) and 2-amino-4-methylpyridinium 4-aminobenzoate (Shen *et al.*, 2008) have been reported. We have recently reported the crystal structures of 2-amino-4-methylpyridinium 4-nitrobenzoate (Hemamalini & Fun, 2010*a*) and 2-Amino-4-methylpyridinium trifluoroacetate (Hemamalini & Fun, 2010*b*) from our laboratory. In continuation of our studies of pyridinium derivatives, the crystal structure determination of the title salt has been undertaken.

The asymmetric unit of the title compound, (Fig 1), contains a protonated 2-amino-4-methylpyridinium cation and a 3-chlorobenzoate anion. The 2-amino-4-methylpyridinium cation is planar, with a maximum deviation of 0.010 (1) Å for atom C1. The protonated N1 atom has lead to a slight increase in the C1—N1—C5 angle to 121.66 (11)°, compared to the corresponding angle of 117.3 (1)° in neutral 2-amino-4-methylpyridine (Kvick & Noordik, 1977). The bond lengths (Allen *et al.*, 1987) and angles are normal.

In the crystal packing, (Fig. 2), the protonated N atom and 2-amino group (N2) is hydrogen-bonded to the carboxylate oxygen atoms (O1 and O2) via a pair of N—H···O hydrogen bonds leading to the formation of a *R*^2^_2_(8) ring (Bernstein *et al.*, 1995).  Furthermore, these motifs are connected via N2—H2C···O2 and C5—H5A···O1 hydrogen bonds to form two-dimensional networks parallel to the *bc*-plane.

### Experimental

A hot methanol solution (20 ml) of 2-amino-4-methylpyridine (54 mg, Aldrich) and 3-chlorobenzoic acid (78 mg, Merck) were mixed and warmed over a heating magnetic-stirrer hotplate for a few minutes. The resulting solution was allowed to cool slowly at room temperature and colourless needles of (I) appeared after a few days.

### Refinement

All hydrogen atoms were positioned geometrically [C–H = 0.93 or 0.96 Å] and were refined using a riding model, with *U*_iso_(H) = 1.2  or 1.5 *U*_eq_(C).  A rotating group model was used for the methyl group. 1860 Friedel pairs were used to determine the absolute configuration.

### Crystal data

**Table:**

C_6_H_9_N_2_^+^·C_7_H_4_ClO_2_^−^	*F*(000) = 276
*M**_r_* = 264.70	*D*_x_ = 1.432 Mg m^−^^3^
Monoclinic, *P*2_1_	Mo *K*α radiation, λ = 0.71073 Å
Hall symbol: P 2yb	Cell parameters from 6601 reflections
*a* = 7.9930 (6) Å	θ = 3.9–35.1°
*b* = 6.8608 (5) Å	µ = 0.31 mm^−^^1^
*c* = 11.2148 (9) Å	*T* = 100 K
β = 93.526 (2)°	Needle, colourless
*V* = 613.84 (8) Å^3^	0.28 × 0.17 × 0.10 mm
*Z* = 2	

### Data collection

**Table:**

Bruker APEXII DUO CCD diffractometer	4207 independent reflections
Radiation source: fine-focus sealed tube	4076 reflections with *I* > 2σ(*I*)
graphite	*R*_int_ = 0.019
φ and ω scans	θ_max_ = 32.5°, θ_min_ = 3.9°
Absorption correction: multi-scan (*SADABS*; Bruker, 2009)	*h* = −12→12
*T*_min_ = 0.919, *T*_max_ = 0.971	*k* = −10→10
9325 measured reflections	*l* = −16→16

### Refinement

**Table:**

Refinement on *F*^2^	Secondary atom site location: difference Fourier map
Least-squares matrix: full	Hydrogen site location: inferred from neighbouring sites
*R*[*F*^2^ > 2σ(*F*^2^)] = 0.029	H-atom parameters constrained
*wR*(*F*^2^) = 0.117	*w* = 1/[σ^2^(*F*_o_^2^) + (0.0801*P*)^2^] where *P* = (*F*_o_^2^ + 2*F*_c_^2^)/3
*S* = 1.22	(Δ/σ)_max_ < 0.001
4207 reflections	Δρ_max_ = 0.64 e Å^−^^3^
164 parameters	Δρ_min_ = −0.54 e Å^−^^3^
1 restraint	Absolute structure: Flack (1983), 1860 Friedel pairs
Primary atom site location: structure-invariant direct methods	Flack parameter: −0.01 (4)

### Special details

**Table:**

Experimental. The crystal was placed in the cold stream of an Oxford Cryosystems Cobra open-flow nitrogen cryostat (Cosier & Glazer, 1986) operating at 100.0 (1) K.
Geometry. All s.u.'s (except the s.u. in the dihedral angle between two l.s. planes) are estimated using the full covariance matrix. The cell s.u.'s are taken into account individually in the estimation of s.u.'s in distances, angles and torsion angles; correlations between s.u.'s in cell parameters are only used when they are defined by crystal symmetry. An approximate (isotropic) treatment of cell s.u.'s is used for estimating s.u.'s involving l.s. planes.
Refinement. Refinement of F^2^ against ALL reflections. The weighted R-factor wR and goodness of fit S are based on F^2^, conventional R-factors R are based on F, with F set to zero for negative F^2^. The threshold expression of F^2^ > 2σ(F^2^) is used only for calculating R-factors(gt) etc. and is not relevant to the choice of reflections for refinement. R-factors based on F^2^ are statistically about twice as large as those based on F, and R- factors based on ALL data will be even larger.

### Fractional atomic coordinates and isotropic or equivalent isotropic displacement parameters (Å^2^)

**Table:**

	*x*	*y*	*z*	*U*_iso_*/*U*_eq_	
Cl1	0.03584 (4)	1.13218 (6)	0.91629 (3)	0.02460 (10)	
O1	0.36548 (12)	0.41445 (16)	0.66474 (7)	0.01744 (18)	
O2	0.36855 (12)	0.47553 (16)	0.86099 (7)	0.01876 (19)	
C7	0.18473 (15)	0.7570 (2)	0.61091 (10)	0.0158 (2)	
H7A	0.2131	0.6782	0.5478	0.019*	
C8	0.09543 (15)	0.9288 (2)	0.58796 (11)	0.0197 (2)	
H8A	0.0654	0.9648	0.5096	0.024*	
C9	0.05103 (16)	1.0466 (2)	0.68188 (12)	0.0195 (2)	
H9A	−0.0078	1.1618	0.6671	0.023*	
C10	0.09626 (15)	0.9890 (2)	0.79852 (10)	0.0159 (2)	
C11	0.18604 (14)	0.8195 (2)	0.82290 (10)	0.0148 (2)	
H11A	0.2155	0.7838	0.9014	0.018*	
C12	0.23182 (14)	0.70259 (19)	0.72825 (10)	0.01258 (19)	
C13	0.32902 (14)	0.51628 (19)	0.75345 (10)	0.0131 (2)	
N1	0.53373 (13)	1.07928 (17)	0.70756 (8)	0.01350 (18)	
H1A	0.4786	1.1863	0.6979	0.016*	
N2	0.53701 (13)	1.1268 (3)	0.91147 (8)	0.0185 (2)	
H2B	0.4837	1.2344	0.8986	0.022*	
H2C	0.5638	1.0902	0.9835	0.022*	
C1	0.57797 (14)	1.01721 (19)	0.82012 (10)	0.0133 (2)	
C2	0.66378 (14)	0.8378 (2)	0.83484 (10)	0.0144 (2)	
H2A	0.6936	0.7919	0.9112	0.017*	
C3	0.70348 (14)	0.73057 (19)	0.73661 (10)	0.0141 (2)	
C4	0.65921 (14)	0.8046 (2)	0.62099 (10)	0.0152 (2)	
H4A	0.6878	0.7364	0.5535	0.018*	
C5	0.57458 (14)	0.9762 (2)	0.60956 (9)	0.0143 (2)	
H5A	0.5441	1.0239	0.5337	0.017*	
C6	0.79035 (16)	0.5371 (2)	0.75110 (12)	0.0195 (2)	
H6A	0.9016	0.5472	0.7239	0.029*	
H6B	0.7286	0.4404	0.7048	0.029*	
H6C	0.7964	0.5002	0.8338	0.029*	

### Atomic displacement parameters (Å^2^)

**Table:**

	*U*^11^	*U*^22^	*U*^33^	*U*^12^	*U*^13^	*U*^23^
Cl1	0.02597 (15)	0.02386 (18)	0.02371 (15)	0.00755 (12)	−0.00058 (10)	−0.00950 (12)
O1	0.0271 (4)	0.0139 (4)	0.0114 (3)	0.0054 (3)	0.0016 (3)	−0.0014 (3)
O2	0.0299 (4)	0.0157 (5)	0.0104 (3)	0.0044 (4)	−0.0002 (3)	0.0000 (3)
C7	0.0166 (4)	0.0184 (6)	0.0123 (4)	0.0012 (4)	0.0013 (3)	0.0016 (4)
C8	0.0203 (5)	0.0227 (7)	0.0160 (5)	0.0047 (5)	0.0006 (4)	0.0050 (5)
C9	0.0189 (5)	0.0184 (7)	0.0213 (5)	0.0041 (4)	0.0007 (4)	0.0019 (5)
C10	0.0147 (4)	0.0158 (6)	0.0173 (4)	0.0005 (4)	0.0016 (3)	−0.0023 (4)
C11	0.0159 (4)	0.0148 (6)	0.0135 (4)	0.0000 (4)	0.0007 (3)	−0.0009 (4)
C12	0.0131 (4)	0.0126 (5)	0.0121 (4)	−0.0009 (4)	0.0013 (3)	0.0007 (4)
C13	0.0175 (4)	0.0108 (5)	0.0109 (4)	−0.0016 (4)	0.0013 (3)	0.0000 (4)
N1	0.0177 (4)	0.0123 (5)	0.0106 (4)	−0.0005 (3)	0.0009 (3)	0.0017 (3)
N2	0.0292 (5)	0.0162 (5)	0.0100 (4)	0.0049 (4)	0.0009 (3)	−0.0003 (4)
C1	0.0164 (4)	0.0130 (5)	0.0104 (4)	−0.0015 (4)	0.0017 (3)	0.0018 (4)
C2	0.0175 (4)	0.0135 (6)	0.0123 (4)	0.0005 (4)	0.0012 (3)	0.0027 (4)
C3	0.0138 (4)	0.0133 (6)	0.0152 (4)	−0.0010 (4)	0.0016 (3)	0.0007 (4)
C4	0.0161 (4)	0.0164 (6)	0.0130 (4)	−0.0008 (4)	0.0012 (3)	−0.0014 (4)
C5	0.0169 (4)	0.0161 (6)	0.0099 (4)	−0.0021 (4)	0.0012 (3)	0.0001 (4)
C6	0.0199 (5)	0.0158 (6)	0.0228 (5)	0.0032 (4)	0.0024 (4)	0.0010 (4)

### Geometric parameters (Å, °)

**Table:**

Cl1—C10	1.7383 (13)	N1—H1A	0.8600
O1—C13	1.2643 (14)	N2—C1	1.3280 (18)
O2—C13	1.2593 (14)	N2—H2B	0.8600
C7—C8	1.3934 (19)	N2—H2C	0.8600
C7—C12	1.3972 (16)	C1—C2	1.4138 (18)
C7—H7A	0.9300	C2—C3	1.3779 (16)
C8—C9	1.3909 (19)	C2—H2A	0.9300
C8—H8A	0.9300	C3—C4	1.4170 (16)
C9—C10	1.3927 (17)	C3—C6	1.5019 (19)
C9—H9A	0.9300	C4—C5	1.3599 (18)
C10—C11	1.3849 (19)	C4—H4A	0.9300
C11—C12	1.3968 (17)	C5—H5A	0.9300
C11—H11A	0.9300	C6—H6A	0.9600
C12—C13	1.5134 (18)	C6—H6B	0.9600
N1—C1	1.3582 (14)	C6—H6C	0.9600
N1—C5	1.3636 (15)		
			
C8—C7—C12	120.35 (12)	C1—N2—H2B	120.0
C8—C7—H7A	119.8	C1—N2—H2C	120.0
C12—C7—H7A	119.8	H2B—N2—H2C	120.0
C9—C8—C7	120.21 (11)	N2—C1—N1	118.49 (12)
C9—C8—H8A	119.9	N2—C1—C2	122.93 (11)
C7—C8—H8A	119.9	N1—C1—C2	118.57 (11)
C8—C9—C10	118.87 (12)	C3—C2—C1	120.36 (10)
C8—C9—H9A	120.6	C3—C2—H2A	119.8
C10—C9—H9A	120.6	C1—C2—H2A	119.8
C11—C10—C9	121.68 (12)	C2—C3—C4	118.91 (11)
C11—C10—Cl1	119.30 (9)	C2—C3—C6	120.86 (11)
C9—C10—Cl1	119.01 (10)	C4—C3—C6	120.22 (11)
C10—C11—C12	119.26 (11)	C5—C4—C3	119.42 (11)
C10—C11—H11A	120.4	C5—C4—H4A	120.3
C12—C11—H11A	120.4	C3—C4—H4A	120.3
C11—C12—C7	119.63 (12)	C4—C5—N1	121.04 (11)
C11—C12—C13	119.90 (10)	C4—C5—H5A	119.5
C7—C12—C13	120.47 (11)	N1—C5—H5A	119.5
O2—C13—O1	125.07 (12)	C3—C6—H6A	109.5
O2—C13—C12	117.52 (10)	C3—C6—H6B	109.5
O1—C13—C12	117.41 (10)	H6A—C6—H6B	109.5
C1—N1—C5	121.66 (11)	C3—C6—H6C	109.5
C1—N1—H1A	119.2	H6A—C6—H6C	109.5
C5—N1—H1A	119.2	H6B—C6—H6C	109.5
			
C12—C7—C8—C9	−0.62 (19)	C11—C12—C13—O1	179.10 (11)
C7—C8—C9—C10	−0.5 (2)	C7—C12—C13—O1	0.27 (16)
C8—C9—C10—C11	0.9 (2)	C5—N1—C1—N2	178.68 (11)
C8—C9—C10—Cl1	−178.18 (10)	C5—N1—C1—C2	−2.06 (17)
C9—C10—C11—C12	−0.32 (18)	N2—C1—C2—C3	−179.79 (12)
Cl1—C10—C11—C12	178.81 (9)	N1—C1—C2—C3	0.98 (17)
C10—C11—C12—C7	−0.79 (17)	C1—C2—C3—C4	0.97 (17)
C10—C11—C12—C13	−179.62 (10)	C1—C2—C3—C6	−178.29 (10)
C8—C7—C12—C11	1.26 (18)	C2—C3—C4—C5	−1.91 (17)
C8—C7—C12—C13	−179.91 (11)	C6—C3—C4—C5	177.35 (11)
C11—C12—C13—O2	−1.50 (17)	C3—C4—C5—N1	0.90 (17)
C7—C12—C13—O2	179.67 (11)	C1—N1—C5—C4	1.13 (17)

### Hydrogen-bond geometry (Å, °)

**Table:**

*D*—H···*A*	*D*—H	H···*A*	*D*···*A*	*D*—H···*A*
N1—H1A···O1^i^	0.86	1.83	2.6921 (16)	175
N2—H2B···O2^i^	0.86	1.93	2.786 (2)	177
N2—H2C···O2^ii^	0.86	1.96	2.8146 (14)	173
C5—H5A···O1^iii^	0.93	2.50	3.1707 (13)	129

Symmetry codes: (i) *x*, *y*+1, *z*; (ii) −*x*+1, *y*+1/2, −*z*+2; (iii) −*x*+1, *y*+1/2, −*z*+1.

## References

[bb1] Aakeroÿ, C. B., Beatty, A. M. & Helfrich, B. A. (2001). *Angew. Chem. Int. Ed.* **40**, 3240–3242. 10.1002/1521-3773(20010903)40:17<3240::AID-ANIE3240>3.0.CO;2-X 29712056

[bb2] Allen, F. H., Kennard, O., Watson, D. G., Brammer, L., Orpen, A. G. & Taylor, R. (1987). *J. Chem. Soc. Perkin Trans. 2*, pp. S1–19.

[bb3] Bernstein, J., Davis, R. E., Shimoni, L. & Chang, N.-L. (1995). *Angew. Chem. Int. Ed. Engl.* **34**, 1555–1573.

[bb4] Bruker (2009). *APEX2*, *SAINT* and *SADABS* Bruker AXS Inc., Madison, Wisconsin, USA.

[bb5] Cosier, J. & Glazer, A. M. (1986). *J. Appl. Cryst.* **19**, 105–107.

[bb6] Flack, H. D. (1983). *Acta Cryst.* A**39**, 876–881.

[bb7] Hemamalini, M. & Fun, H.-K. (2010*a*). *Acta Cryst.* E**66**, o335. 10.1107/S1600536810000693 PMC2979821 21579764

[bb8] Hemamalini, M. & Fun, H.-K. (2010*b*). *Acta Cryst.* E**66**, o781–o782. 10.1107/S1600536810008202 PMC2984062 21580622

[bb9] Jeffrey, G. A. (1997). *An Introduction to Hydrogen Bonding.* Oxford University Press.

[bb10] Jeffrey, G. A. & Saenger, W. (1991). *Hydrogen Bonding in Biological Structures.* Berlin: Springer.

[bb11] Kvick, Å. & Noordik, J. (1977). *Acta Cryst.* B**33**, 2862–2866.

[bb12] Remenar, J. F., Morissette, S. L., Peterson, M. L., Moulton, B., MacPhee, J. M., GuzmaÅ, H. R. & Almarsson, O. È. (2003). *J. Am. Chem. Soc.* **125**, 8456–8457. 10.1021/ja035776p 12848550

[bb13] Scheiner, S. (1997). *Hydrogen Bonding. A Theoretical Perspective.* Oxford University Press.

[bb14] Sheldrick, G. M. (2008). *Acta Cryst.* A**64**, 112–122. 10.1107/S0108767307043930 18156677

[bb15] Shen, H., Nie, J.-J. & Xu, D.-J. (2008). *Acta Cryst.* E**64**, o1129. 10.1107/S1600536808014839 PMC2961432 21202640

[bb16] Sokolov, A. N., FrisïcïcÅ, T. & MacGillivray, L. R. (2006). *J. Am. Chem. Soc.* **128**, 2806–2807. 10.1021/ja057939a 16506752

[bb17] Spek, A. L. (2009). *Acta Cryst.* D**65**, 148–155. 10.1107/S090744490804362X PMC2631630 19171970

